# Constraints on Knot Insertion, Not Internal Jamming, Control Polycatenane Translocation Dynamics through Crystalline Pores

**DOI:** 10.1021/acs.macromol.2c02565

**Published:** 2023-04-10

**Authors:** Zifeng Wang, Robert M. Ziolek, Mesfin Tsige

**Affiliations:** † School of Polymer Science and Polymer Engineering, The University of Akron, Akron, Ohio 44325-3909, United States; ‡ Biological Physics and Soft Matter Group, Department of Physics, King’s College London, London WC2R 2LS, United Kingdom

## Abstract

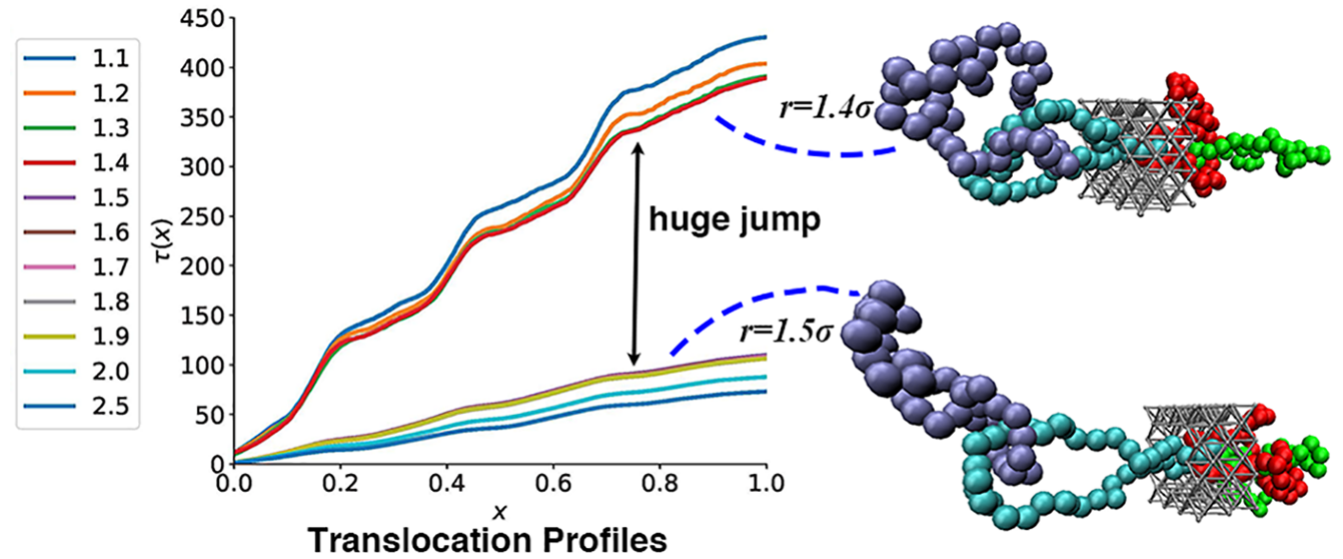

The translocation of polymers through pores and channels is an archetypal process in biology and is widely studied and exploited for applications in bio- and nanotechnology. In recent times, the translocation of polymers of various different topologies has been studied both experimentally and by computer simulation. However, in some cases, a clear understanding of the precise mechanisms that drive their translocation dynamics can be challenging to derive. Experimental methods are able to provide statistical details of polymer translocation, but computer simulations are uniquely placed to uncover a finer level of mechanistic understanding. In this work, we use high-throughput molecular simulations to reveal the importance that knot insertion rates play in controlling translocation dynamics in the small pore limit, where unexpected nonpower law behavior emerges. This work both provides new predictive understanding of polycatenane translocation and shows the importance of carefully considering the role of the definition of translocation itself.

## Introduction

The translocation of polymers through pores is ubiquitous in biology, and it is driving new advances in contemporary nano- and biotechnology.^1−3^ In biological settings, translocation processes include the passage of mRNA and DNA through pore complexes^4^ and the transport of proteins through membrane channels, including during viral infection.^5^ Polymer translocation also has broad practical applications in biotechnology, spanning polymer characterization and separation,^6,7^ gene therapy,^8^ DNA sequencing,^9,10^ and controlled drug delivery.^11^ Since the pioneering works initiated the polymer translocation research field nearly three decades ago,^12,13^ experimental research has been underpinned by a wide range of theoretical^14−20^ and simulation-based studies.^21−41^

Early computer simulation investigations of polymer translocation focused primarily on linear polymers. It was demonstrated for unforced translocation, i.e., in the absence of an external driving force, that the average translocation time τ typically follows the power law τ ∼ *N*^α^ in which *N* is the chain length and the magnitude of α indicates the ease of the polymer translocation process.^14,16,21,22^ With the addition of an external driving force with amplitude *f*, the scaling behavior changes to τ ∼ *N*^α^*f*^–δ^, where the coefficient δ accounts for the impact of the applied force on the translocation. More recently, the translocation of polymers with different topologies has become an active research area. While the translocation of ring polymers has been shown to be only quantitatively different with respect to analogous linear polymers of the same length,^32,33,38^ other polymer topologies show more complex behavior.^39,40^ It has been shown that two different modes of translocation exist for the knotted regions of DNA^36^ and that the number of arms in a star polymer directly controls its translocation dynamics.^41^

Catenanes are a mechanically interlocked molecular architecture consisting of two mechanically interlocked macrocycles, the first synthesis of which in 1983 led to the award of part of the 2016 Nobel Prize in Chemistry.^42^ Polycatenanes, which are formed of three or more consecutively interlocked macrocycles, are finding use in a variety of new advanced technologies, such as molecular motors and switches.^43^ More recently, simulations and experiments have been used to show that a topological obstruction in catenanes dramatically slows their translocation dynamics through a pore,^34,44^ while the dependence on pore size has not yet been considered. In this work, we determine the pore size-dependent mechanism of the translocation of a poly[4]catenane (hence polycatenane) using molecular dynamics simulations. By simulating over a range of pore sizes smaller than those previously considered in the literature, we enter a regime where pore microstructure effects become significant. We show that an unexpected jump in translocation time occurs at a critical pore radius and that this effect is universal for both the polycatenane and an analogous ring polymer. The translocation time jump is more pronounced for the polycatenane; by performing a large number of replica simulations we are able to uncover the underlying mechanism behind this observation, which is not a result of chain jamming within the pore. In doing this, we show that the number of beads within the pore does not correlate with the instantaneous waiting time, which implies the number of beads within the pore may not be a good indicator variable when studying polymer translocation. This new understanding comes about because of a thorough treatment of the translocation definition itself, which is an often overlooked detail. We show the translocation definition has a nontrivial effect on the final translocation profiles, which can be rationalized by considering the underlying mathematical details of the different definitions. Using this framework, we uncover new insights regarding recently reported experimental and simulation results previously reported in the literature. This work therefore provides new predictive understanding that can be applied to the development of new molecular technologies that make use of polymer translocation.

## Model and Methods

### Simulation Details

The pore is modeled as a cylindrical aperture of radius *r* within a FCC slab measuring 20σ × 20σ × 4σ (4σ is therefore the length of the pore). A total of 11 different pore radii are considered ranging from *r* = 1.1σ to *r* = 2.0σ in steps of 0.1σ as well as a further system with the larger pore size of *r* = 2.5σ. In our simulations, we consider a poly[4]catenane (consisting of 4 mechanically interlocked rings of 40 beads each) as well as a simple ring polymer consisting of 160 beads for reference. We use massive, rather than massless, beads as per various other studies.^34,45,46^ We employ Langevin dynamics to model polymer translocation with a constant external pulling force on each bead inside the pore, as described by
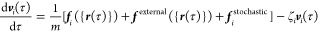
1where *m* is the mass of each bead. We model polymer chains using a coarse-grained bead–spring model. Bonds between connected beads are modeled using the finite extension nonlinear elastic (FENE) potential
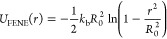
2where *k*_b_ = 30*ϵσ*^–2^ is the bond spring constant, *R*_0_ = 1.5σ is the maximum bond length, and *r* is the bond length. A short-range repulsive Lennard–Jones potential is used to prevent overlap between beads, modeling interactions between the polymer beads and the pore as
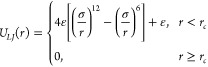
3where *r*_*c*_ = 2^1/6^σ, such that the potential is purely repulsive. In the present work, we use the Lennard–Jones parameters, ε and σ, and the monomer mass, *m*, to fix the energy length and mass scales, respectively. A time step of Δ*t* = 0.0025τ was selected, where  is the characteristic simulation time. The external driving force, applied to all of the beads inside the pore, ***f***^external^, has magnitude |***f***^external^| = *ϵσ*^–1^.^28^ Our simulation methodology does not account for hydrodynamic interactions (HI) through the inclusion of explicit solvent beads: the effect of HI upon polymer translocation through small pores has been shown to be negligible using both molecular dynamics and lattice Boltzmann simulations.^47,48^

**Figure 1:**
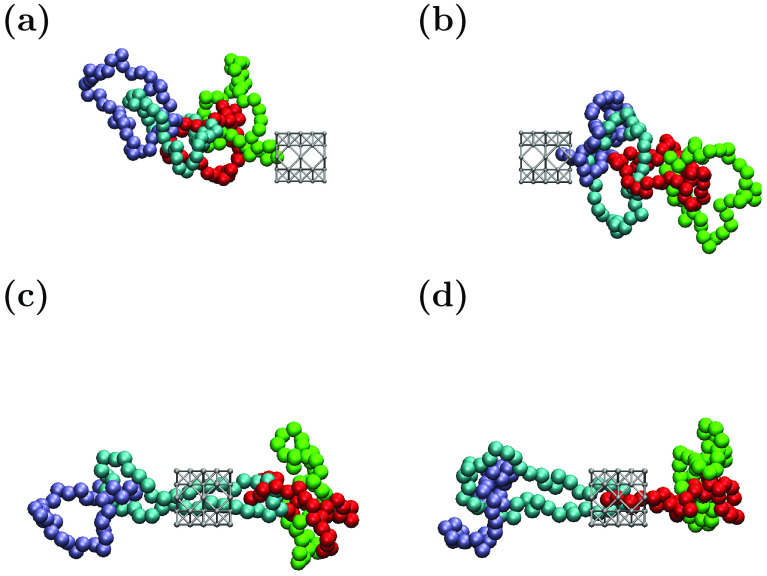
Simulation snapshots showing (a) the initial (left) and (b) the final (right) states of the polycatenane from a production simulation, (c) part of the simulation run with a single ring within the pore, and (d) a knot (Hopf link) passing through the pore during the simulation. The four polycatenane rings are shown in different colors, and a small part of the FCC pore structure is shown in gray.

The initial polycatenane conformation for each production simulation, as exemplified in Figure 1a, is obtained by fixing one bead of the polymer at the entrance of the pore and then allowing the rest of the chain to evolve until reaching equilibrium, as monitored through the evolution and subsequent equilibration of its radius of gyration (Figure S1). Production simulations were then conducted until the polymer fully translocated through the pore under the influence of the external driving force. For each system corresponding to a given pore radius, we performed 140 independent replica simulations beginning from a random initial polymer conformation for both the polycatenane and the ring polymers. Therefore, this manuscript reports on the results from a total of 3080 independent simulations. All simulations were conducted using the LAMMPS simulation engine.^49^

### Analysis Techniques

Simulation analysis was performed using codes developed in house, which make wide use of the MDAnalysis and MDTraj Python packages.^50,51^ Simulation visualizations were produced with VMD.^52^

#### Tracking Polymer Translocation

One definition, the irreversible translocation fraction (*x*^irrev^(τ)), measures the time at which each bead in the polycatenane passes through the pore entrance located at position *x*_*p*_ on the translocation axis. For a given bead *i*, its translocation time τ_*i*_ is
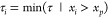
4From this, we can determine the translocation time (τ) as a function of the translocation fraction to yield our final result for *x*^irrev^(τ)
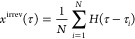
5where *H*(τ – τ_*i*_) is a Heaviside function
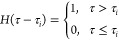
6This definition of *x*^irrev^(τ) dictates that it is an increasing function since once a given bead is determined to have crossed the pore, any instantaneous backward translocation will not be accounted for. Alternatively, we can measure the translocation fraction directly at each τ as to yield a reversible translocation fraction *x*^rev^(τ) as
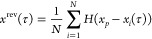
7where *H*(*x*_*p*_ – *x*_*i*_(τ)) is again a Heaviside function
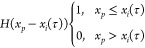
8This second approach accounts for fluctuations in the chain position during the translocation process.

#### Measuring Polymer Orientation

We use the following order parameter to assess the overall orientation of the polymer ring(s) within the pore interior
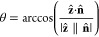
9where **ẑ** is the unit vector in the *z* direction (i.e., perpendicular to the pore translocation axis, *x*) and **n̂** is the normal vector of the best-fitting surface of the beads of the polycatenane within the pore. **n̂** is calculated using singular value decomposition (SVD), which finds the most probable two-dimensional plane that fits to the bead positions within the pore.

#### Entropy of a Probability Density Distribution

The Shannon entropy of a discrete probability distribution *p*_*X*_ is calculated as
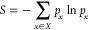
10where *p*_*x*_ is the probability at bin *x*. For a discrete probability density distribution ρ_*X*_, note that *p*_*x*_ = ρ_*x*_/Σ_*x*∈*X*_ρ_*x*_.

Note that *S* is maximized over a given domain (max(*S*) = ln(N)) when the underlying probability distribution is the uniform distribution of length N. We consider the symmetry of the pores studied as required during the different analyses.

## Results and Discussion

### Two Definitions Yield Contrasting Pictures of Translocation

**Figure 2:**
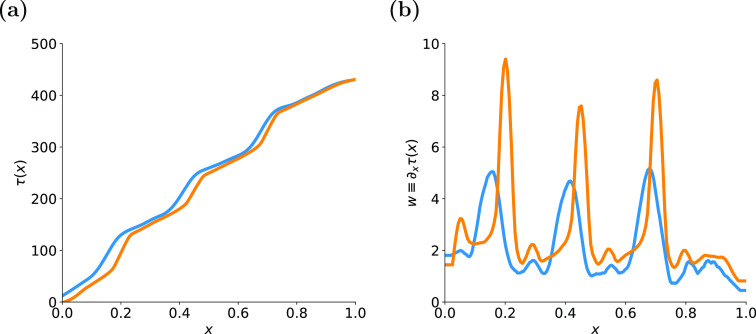
Understanding the differences of the two translocation definitions. Results from simulations with *r* = 1.1 clearly highlight the differences between the irreversible (orange) and the reversible (blue) definitions of translocation. (a) Translocation time as a function of translocation fraction. (b) Waiting time as a function of translocation fraction. The Savitzky–Golay digital filter has been applied to the data.^53^

Translocation (*x*) may reasonably be defined in two ways (see Methods and Model). The two approaches yield rather different translocation profiles for the same set of simulation trajectories. Figure 2a shows the translocation profiles averaged from multiple simulations for pore radius *r* = 1.1 obtained by both translocation definitions. Note that the time (τ) associated with translocation progress is always shorter for the irreversible translocation definition (i.e., τ(*x*^irrev^) ≤ τ(*x*^rev^) *∀ τ*). The total time for translocation for each definition remains identical however. During the parts of the translocation roughly corresponding to when the polycatenane knots pass through the pore, a slowdown in translocation is indicated by the increase in the instantaneous waiting time in Figure 2b; this feature will be explored in detail later in the manuscript. The waiting time peaks in Figure 2b are greater and narrower for the irreversible translocation definition (orange line) than for those for the reversible translocation definition (blue line). Additionally, the peaks associated with the reversible translocation definition have their corresponding maxima located at slightly lower values of *x*.

Since the irreversible translocation definition does not account for the underlying fluctuations in the translocation process, the sharp peaks in the waiting time are considered to be artificially large and not truly representative of the system evolution. Using the reversible translocation description, one infers that the polymer knot translocation actually occurs over a longer portion of the whole translocation process (i.e., yielding a broader peak waiting time). Furthermore, the minimum polycatenane translocation rate is greater than that described by the irreversible translocation definition (corresponding to the lower waiting time peak maximum values). These results highlight the importance of using a translocation definition that can account for the stochastic fluctuations that are present in the translocation process. As such, we exclusively use the reversible translocation definition throughout the remainder of this work.

### Polycatenane Translocation Dynamics Are Not Proportional to the Pore Radius in the Small Pore Limit

We focus on understanding the effect of the pore radius upon the translocation dynamics of the polycatenane by studying translocation through different crystalline pore sizes, ranging from *r* = 1.1 to *r* = 2.5. The simulation visualizations presented in Figure S2 visually exemplify how the pore microstructure aids the translocation of a Hopf link through the smallest pore size considered in this work (*r* = 1.1). Additionally, the FENE bond potential (eq 2) is defined up to a maximum value of 1.5σ, so both bond deformation and the pore microstructure can act to accommodate Hopf link translocation through the particularly small pores considered in this work. These pore sizes are smaller than those typically studied in the literature.^23,24,34−37,54^ As such, we observe the emergence of atypical dynamics over the range of pore sizes that do not follow the archetypal power law behavior observed over sets of larger pore sizes.

**Figure 3:**
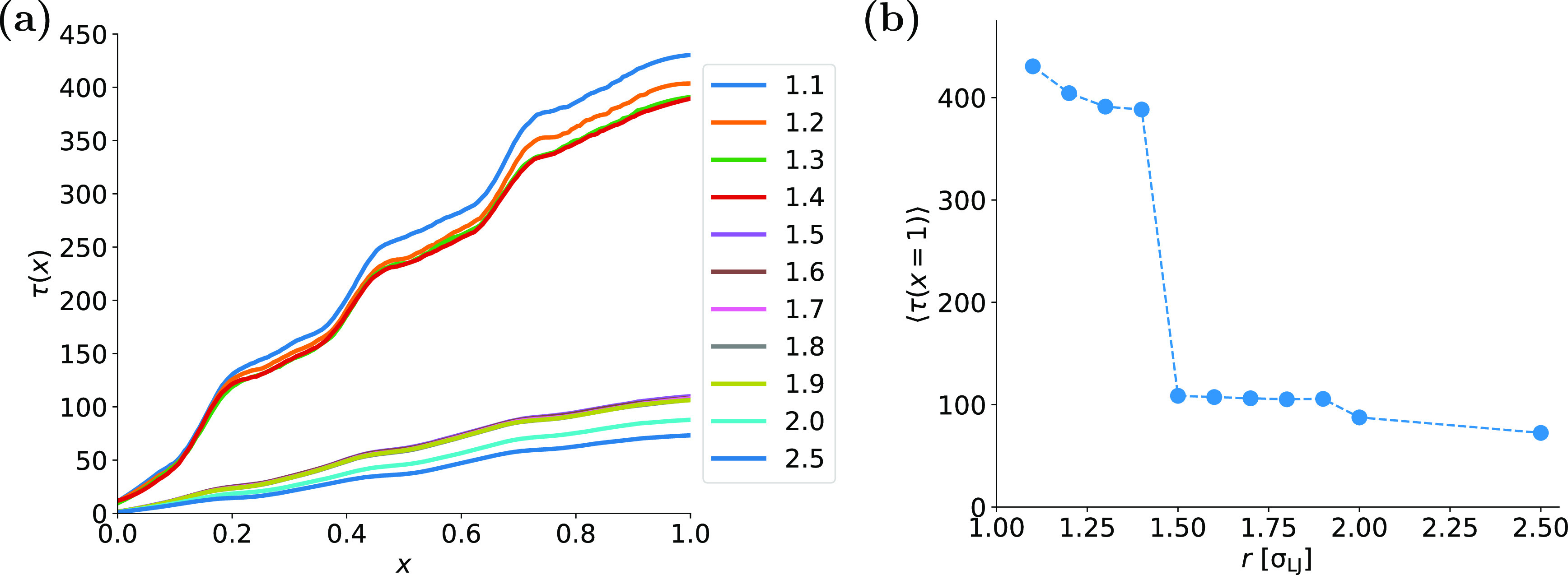
(a) Translocation profiles for a range of pore radii (see the plot sidebar for colors), and (b) corresponding total translocation times for the different systems considered. Error bars representing the 95% confidence interval are smaller than the data point markers.

Figure 3a shows the average translocation profiles for each of the different pore sizes, while Figure 3b shows the average total translocation time for each system. For the smallest pore sizes (i.e., *r* ≤ 1.4), the translocation profiles exhibit the same features with a small decrease in the translocation time observed as *r* is incrementally increased. A large jump in translocation time is then unexpectedly observed between *r* = 1.4 and *r* = 1.5. In the case of the largest pore radius considered here, *r* = 2.5, the translocation profile shows only a slight slowdown in translocation as each of the Hopf links pass through the pore; these features are very similar to the analogous profile of a catenane previously reported by Caraglio et al. for a slightly larger pore radius (*r* = 3).^34^ By considering a range of smaller pore radii, we observe that multiple translocation regimes emerge as a function of the pore radius, which cannot be encapsulated by a power law model. For simulations of an analogous ring polymer consisting of 160 beads, an analogous large jump is observed (see Figure S3a and S3b). This indicates that the jump in translocation time is not driven by the topology of the polycatenane alone, although the difference in translocation rate around this jump point is greater for the polycatenane than the ring polymer (by a factor of approximately 4, rather than 3). This universality has not previously been demonstrated in the small pore size limit. We note also that the jump is also observed to occur at the same critical pore radius for the polycatenane when placed under a greater external force, i.e., |***f***^external^| = 2*ϵσ*^–1^ (Figure S4).

**Figure 4:**
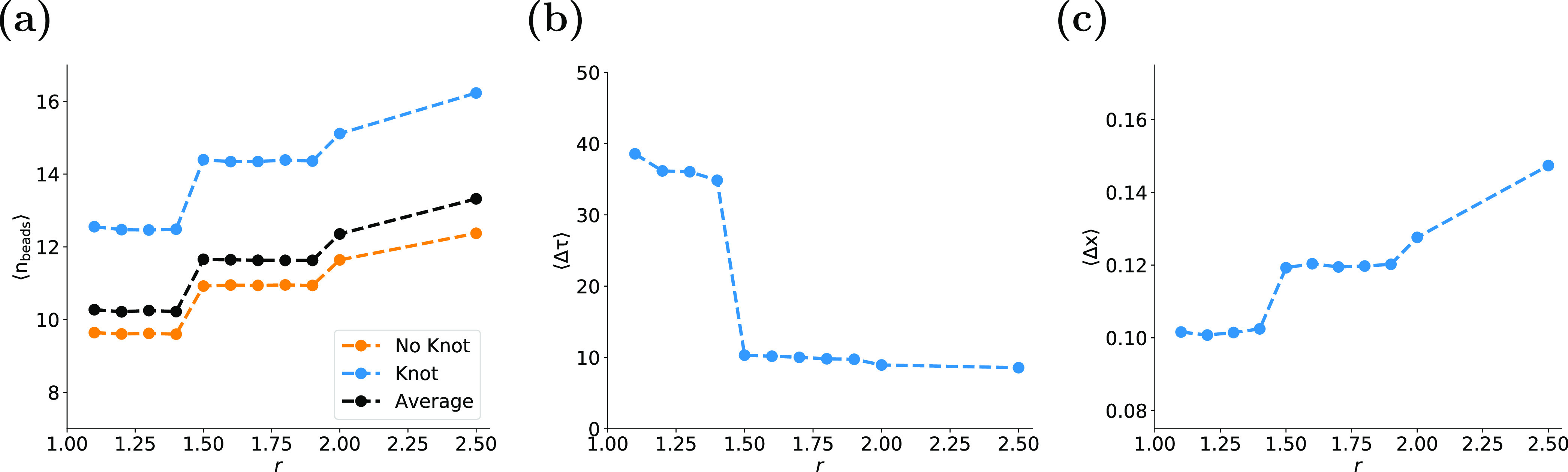
(a) Number of beads within the pore as a function of the pore radius. (b) Average time taken for knot passage events as a function of the pore radius. (c) Average translocation fraction associated with knot passage events as a function of the pore radius. Error bars representing the 95% confidence interval are smaller than the data point markers in all cases.

To understand the translocation of the polycatenane as a function of the pore radius, we investigated the statistical properties of the knot passage events during translocation. Figure 4a shows the average number of polycatenane beads found within the pore over the entire translocation process (black) and when a knot is (or is not) present in the pore during translocation. As anticipated, we see that there are more beads in the pore when the knot is passing through, and the average number of beads in the pore is therefore slightly higher than that for the corresponding single-ring polymer system (see Figure S3c). Reflecting the large jump in the translocation time moving from *r* = 1.4 to *r* = 1.5 is the corresponding increase in the average number of beads found within the pore. The comparatively small relative increase in the number of beads within the pore itself does not account for the corresponding large drop in translocation time. Furthermore, the average time taken for the polycatenane knots to pass through the pore, ⟨Δτ⟩, is shown in Figure 4b, and the corresponding average translocation fraction, ⟨Δ*x*⟩, for knot passage is shown in Figure 4c. The difference in Δτ between *r* = 1.4 and *r* = 1.5 is less than 30τ, so the difference in total knot translocation time (3 × 30τ = 90τ) cannot alone account for the jump in the overall translocation time (Figure 3b), which is approximately three times as large (∼300τ).

### Geometric Constraint on Knot Insertion Controls Translocation

**Figure 5:**
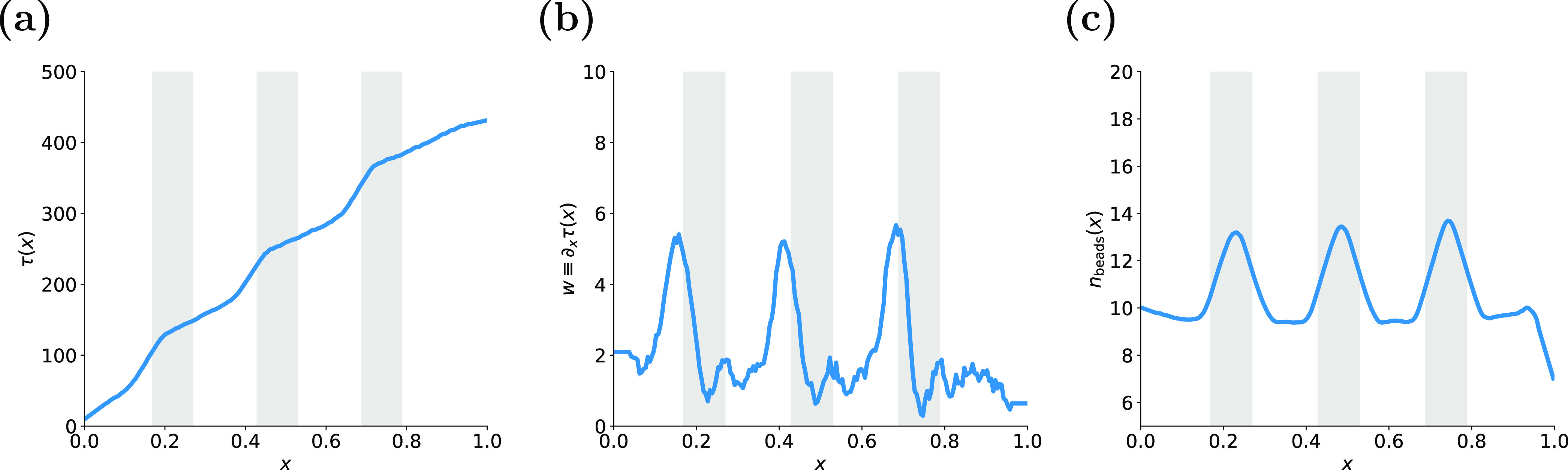
Dynamics of polycatenane translocation in the small pore size limit. Results for simulations with *r* = 1.1. (a) Translocation time (τ) as a function of translocation fraction (*x*). (b) Waiting time as a function of translocation fraction. (c) Number of beads within the pore as a function of translocation fraction. Note that the regions of each plot shaded in gray correspond to the periods of translocation during which the polycatenane knots pass through the pore.

Figure 5 shows the translocation profile, associated waiting time profile, and number of beads within the pore as a function of translocation fraction for polycatenane translocation through the smallest pore size considered here (*r* = 1.1). The analogous results for all of the different pore sizes studied here are shown in the Supporting Information (see Figures S5–S7). It might be expected that the translocation of the polycatenane simply is made up of two simple random walk regimes, one describing the parts of the translocation when a knot is passing through the pore and another when a single ring is passing through. The results presented in this section show that this simple picture is not a suitable description of the polycatenane translocation. Instead, a more complex translocation process with a number of interesting features is observed. This becomes more clear by breaking down a translocation profile and the associated waiting time and instantaneous number of beads within the pore into two parts. In Figure 5, parts of the translocation dynamics where knots are passing through the pore are highlighted by the gray background shading. We automatically identified the passage of knots through the pore as the periods of each trajectory where beads from more than one ring were present within the pore interior.

In the newly annotated translocation profile shown in Figure 5a, a number of regimes now emerge more clearly. Initially, translocation of the polycatenane progresses freely as the first ring within the polycatenane passes through the pore. A slowdown in translocation is then observed as the first knot within the polycatenane approaches the pore, rather than upon its actual entry (this slowdown occurs before the first region of gray shading). As demonstrated in Figure 5b, the peaks in the waiting time occur slightly before the knot itself enters the pore. As a knot enters the pore, there is a clear decrease in waiting time as a function of *x*, which corresponds to faster translocation as the knot itself passes through the pore. We also see that the number of beads within the pore rises slightly immediately before the knot itself enters the pore but rises to its maximum value after the knot actually enters the pore (see Figure 5c), when the translocation rate has increased. As the knots exit the pore, there are small associated local maxima in the waiting time results before they are freed. After the final of the three knots has passed through the pore, there is an increase in the translocation rate. This is an entropic effect: the polymer tail quickly retracts through the pore at the end of the translocation process as the slack in the polycatenane is released.^10,16^ Given that the slowdown of translocation actually occurs in the time immediately preceding the beginning of each knot translocation, understanding the origin of this slowdown will help to uncover the underlying mechanism that controls translocation rates as a function of the pore radius.

We can also now make direct comparison to a recently reported experimental translocation profile of a poly[4]catenane,^44^ where the measured translocation blockade current is inversely proportional to the amount of DNA instantaneously found within a pore (see Figure 2 in ref (44)). In addition to the number of beads within the pore as a function of translocation fraction, as shown in Figure 5c, we present the number of beads within the pore as a function of time for some different simulations in Figure S8. We note the agreement between these simulation-derived results to those reported by Rheaume and Klotz, with the three peaks each of the plots in Figure S8 corresponding inversely to their experimentally measured profile.

While the pores were generated with a specific radius, at such small pore sizes the structure of the pore interior within the FCC crystal is not perfectly cylindrical (see Figure S2). This microscopic pore structure can be demonstrated by considering the probability distributions of the relative orientation that the polycatenane rings adopt while passing through the pore (Figure S9). The changes in pore structure place specific constraints on the polycatenane. In the small pore size limit, the internal structure of the pore is clearly evident: the probability densities of polycatenane orientation (shown in Figure S9a–d) show that the polycatenane is restricted to adopting specific orientations within the pore at all times, not just at knot insertion, whereas this changes before the probability density becomes effectively uniform at large pore sizes.

**Figure 6:**
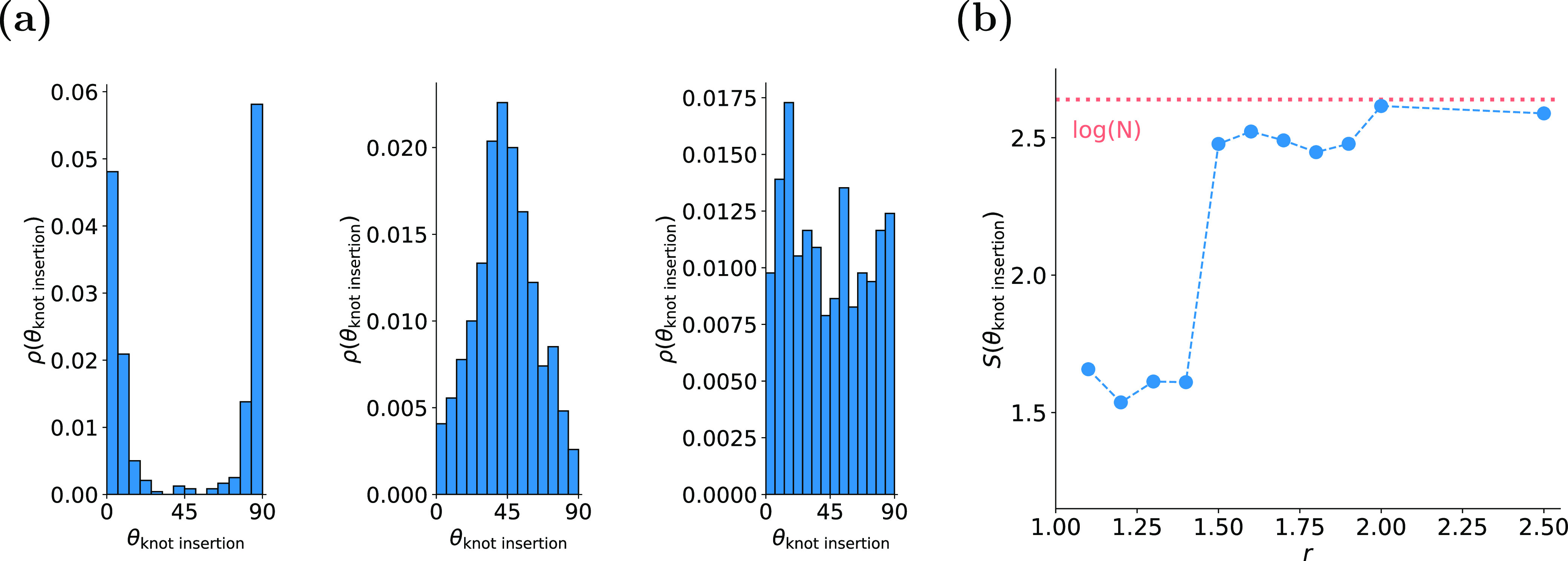
(a) Probability densities of the polycatenane orientation inside the pore at the point of knot insertion for *r* = 1.4, 1.5, and 2.0. (b) Shannon entropy of these probability densities as a function of the pore radius. The maximum entropy of the distributions is shown by the pink line (max(*S*) = log(*N*) for a uniform distribution with length *N*).

Interestingly, the orientation of the polycatenane ring already within the pore at the point of knot entry provides a clear mechanism for the unexpected translocation dynamics as a function of pore size. Figure S10 shows the probability densities of the ring orientation inside the pore at the time of these specific events. After consideration of the symmetry in the probability densities in Figure S9 (which arises due to interactions between the polymer rings and the internal structure of the pore), these distributions are presented such that if θ > 90°; then, a conversion is applied (θ → 90 – θ) to reflect this underlying symmetry and also to reduce noise in the statistics. Figure 6a shows the probability densities of the ring orientation at the point of knot insertion for selected pore sizes.

Note that the distributions for small pore sizes show a more restricted range of angles than the corresponding distributions that describe the insertion angle of the polycatenane at all points of the translocation process rather than just the knot entry events (see Figure S10). This highlights that the polycatenane rings must adopt a very specific orientation in pores with *r* ≤ 1.4 to allow the next ring to enter the pore. This requires significant reorganization of the chains within the pore, which in turn is responsible for the hindered translocation before knot insertion. A wider range of orientation angles is found for *r* ≤ 1.5 (with the most probable value of 45°) as the pore both increases in size and changes shape. For the largest pores (*r* ≥ 2.0), the distributions are essentially uniform, although noise inherent in the distributions prevents the theoretical maximum entropy from being reached. The Shannon entropy of these distributions (Figure 6b) highlights the constraints on chain orientation to allow knot insertion in the smallest pores. A large jump in *S* between *r* = 1.4 and *r* = 1.5 suggests that a greater number of possible orientations becomes available as the pore size increases slightly, which greatly increases the rate of knot insertion into the pore. In turn, this leads to the large drop in total translocation time observed in Figure 3 between *r* = 1.4 and *r* = 1.5.

## Conclusions

This work exemplifies the value of high-throughput molecular simulations in understanding the complex translocation behavior of a model polycatenane. An important feature of our study is indeed the large number of replica simulations performed (140 per pore radius): only with this great amount of data does the underlying mechanism of translocation control clearly emerge from the underlying dynamical stochasticity. Furthermore, care was taken to understand the ramifications of the exact translocation definition used to model the dynamics, which has been often overlooked in the literature. We propose that our reversible translocation definition provides a suitable framework since it accounts for the stochastic fluctuations that are present in the translocation process. These careful technical considerations allowed us to unravel the mechanism that drives the translocation dynamics of the polycatenane through a crystalline pore. Interestingly, considering the number of beads within the pore in isolation did not resolve the pore size effects. The unexpected sudden jump in translocation time can be rationalized by the observation that a greater number of possible orientations become available as the pore size increases slightly from *r* = 1.4 and *r* = 1.5, which greatly increases the rate of knot insertion into the pore. Therefore, the rate of insertion of knots into the pore controls the overall translocation rate, rather than jamming of the knots within the pore itself.

## Supporting Information Available

Polycatenane equilibration; pore microstructure; ring polymer translocation; translocation profiles for different external forces; translocation profiles, waiting time profiles, and profiles showing the number of beads inside the pore; number of beads within the pore as a function of time; polycatenane orientation distributions; polycatenane insertion angle distributions (PDF)

## References

[ref1] Chen K.; Kong J.; Zhu J.; Ermann N.; Predki P.; Keyser U. F. Digital Data Storage Using DNA Nanostructures and Solid-State Nanopores. Nano Lett. 2019, 19, 1210–1215. 10.1021/acs.nanolett.8b04715. 30585490

[ref2] Knowles S. F.; Weckman N. E.; Lim V. J. Y.; Bonthuis D. J.; Keyser U. F.; Thorneywork A. L. Current Fluctuations in Nanopores Reveal the Polymer-Wall Adsorption Potential. Phys. Rev. Lett. 2021, 127, 137801 10.1103/PhysRevLett.127.137801. 34623825

[ref3] Zhang X.; Luo D.; Zheng Y.-W.; Li X.-Q.; Song J.; Zhao W.-W.; Chen H.-Y.; Xu J.-J. Translocation of Specific DNA Nanocarrier through an Ultrasmall Nanopipette: Toward Single-Protein-Molecule Detection with Superior Signal-to-Noise Ratio. ACS Nano 2022, 16, 15108–15114. 10.1021/acsnano.2c06303. 36047811

[ref4] Muthukumar M. Mechanism of DNA transport through pores. Annu. Rev. Biophys. Biomol. Struct. 2007, 36, 435–450. 10.1146/annurev.biophys.36.040306.132622. 17311526

[ref5] Alberts B.; Bray D.; Lewis J.; Raff M.; Roberts K.; Watson J. D. Molecular Biology of the Cell; Garland Science: New York, 1994.

[ref6] Ding M.; Duan X.; Shi T. Flow-induced translocation of star polymers through a nanopore. Soft Matter 2016, 12, 2851–2857. 10.1039/C6SM00040A. 26879130

[ref7] Ge H.; Wu C. Separation of linear and star chains by a nanopore. Macromolecules 2010, 43, 8711–8713. 10.1021/ma101849k.

[ref8] Lu Q.; Moore J. M.; Huang G.; Mount A. S.; Rao A. M.; Larcom L. L.; Ke P. C. RNA polymer translocation with single-walled carbon nanotubes. Nano Lett. 2004, 4, 2473–2477. 10.1021/nl048326j.

[ref9] Kasianowicz J. J.; Brandin E.; Branton D.; Deamer D. W. Characterization of individual polynucleotide molecules using a membrane channel. Proc. Natl. Acad. Sci. U. S. A. 1996, 93, 13770–13773. 10.1073/pnas.93.24.13770. 8943010 PMC19421

[ref10] Han J.; Turner S.; Craighead H. G. Entropic trapping and escape of long DNA molecules at submicron size constriction. Phys. Rev. Lett. 1999, 83, 1688 10.1103/PhysRevLett.83.1688.

[ref11] Chang D. C. Structure and dynamics of electric field-induced membrane pores as revealed by rapid-freezing electron microscopy. Guide to electroporation and electrofusion; Elsevier, 1992; pp 9–27; 10.1016/B978-0-12-168040-4.50005-5.

[ref12] Bezrukov S. M.; Vodyanoy I.; Parsegian V. A. Counting polymers moving through a single ion channel. Nature 1994, 370, 279–281. 10.1038/370279a0. 7518571

[ref13] Kasianowicz J.; Brandin E.; Branton D.; Deamer D. Characterization of individual polynucleotide molecules using a membrane channel. Proc. Natl. Acad. Sci. U. S. A. 1996, 93, 13770–13773. 10.1073/pnas.93.24.13770. 8943010 PMC19421

[ref14] Park P.; Sung W. Polymer translocation through a membrane pore. Phys. Rev. Lett. 1996, 77, 783–786. 10.1103/PhysRevLett.77.783. 10062901

[ref15] Wei C.; Srivastava D. Theory of transport of long polymer molecules through carbon nanotube channels. Phys. Rev. Lett. 2003, 91, 235901 10.1103/PhysRevLett.91.235901. 14683200

[ref16] Muthukumar M. Polymer translocation through a hole. J. Chem. Phys. 1999, 111, 10371–10374. 10.1063/1.480386.

[ref17] Muthukumar M. Translocation of a confined polymer through a hole. Phys. Rev. Lett. 2001, 86, 3188 10.1103/PhysRevLett.86.3188. 11290139

[ref18] Slonkina E.; Kolomeisky A. B. Polymer translocation through a long nanopore. J. Chem. Phys. 2003, 118, 7112–7118. 10.1063/1.1560932.

[ref19] Kong C.; Muthukumar M. Polymer translocation through a nanopore. II. Excluded volume effect. J. Chem. Phys. 2004, 120, 3460–3466. 10.1063/1.1642588. 15268503

[ref20] Park P. J.; Sung W. Polymer translocation induced by adsorption. J. Chem. Phys. 1998, 108, 3013–3018. 10.1063/1.475688.

[ref21] Chuang J.; Kantor Y.; Kardar M. Anomalous dynamics of translocation. Phys. Rev. E 2001, 65, 011802 10.1103/PhysRevE.65.011802. 11800711

[ref22] Luo K.; Ala-Nissila T.; Ying S.-C. Polymer translocation through a nanopore: A two-dimensional Monte Carlo study. J. Chem. Phys. 2006, 124, 034714 10.1063/1.2161189. 16438607

[ref23] Yong H.; Wang Y.; Yuan S.; Xu B.; Luo K. Driven polymer translocation through a cylindrical nanochannel: interplay between the channel length and the chain length. Soft Matter 2012, 8, 2769–2774. 10.1039/c2sm06942c.

[ref24] Nikoofard N.; Khalilian H.; Fazli H. Directed translocation of a flexible polymer through a cone-shaped nano-channel. J. Chem. Phys. 2013, 139, 074901 10.1063/1.4818419. 23968109

[ref25] Mohan A.; Kolomeisky A. B.; Pasquali M. Polymer translocation through pores with complex geometries. J. Chem. Phys. 2010, 133, 024902 10.1063/1.3458821. 20632771

[ref26] Cohen J. A.; Chaudhuri A.; Golestanian R. Stochastic sensing of polynucleotides using patterned nanopores. Phys. Rev. X 2012, 2, 021002 10.1103/PhysRevX.2.021002.

[ref27] Luo K.; Ala-Nissila T.; Ying S.-C.; Bhattacharya A. Sequence dependence of DNA translocation through a nanopore. Phys. Rev. Lett. 2008, 100, 058101 10.1103/PhysRevLett.100.058101. 18352434

[ref28] Niknam Hamidabad M.; Haji Abdolvahab R. Translocation through a narrow pore under a pulling force. Sci. Rep. 2019, 9, 1–12. 10.1038/s41598-019-53935-3. 31784562 PMC6884557

[ref29] Wei D.; Yang W.; Jin X.; Liao Q. Unforced translocation of a polymer chain through a nanopore: The solvent effect. J. Chem. Phys. 2007, 126, 204901 10.1063/1.2735627. 17552794

[ref30] Xie Y.; Yang H.; Yu H.; Shi Q.; Wang X.; Chen J. Excluded volume effect on confined polymer translocation through a short nanochannel. J. Chem. Phys. 2006, 124, 174906 10.1063/1.2195459. 16689603

[ref31] Luan B.; Aksimentiev A. Electro-osmotic screening of the DNA charge in a nanopore. Phys. Rev. E 2008, 78, 021912 10.1103/PhysRevE.78.021912. PMC2887483 18850870

[ref32] Ding M.; Duan X.; Lu Y.; Shi T. Flow-induced ring polymer translocation through nanopores. Macromolecules 2015, 48, 6002–6007. 10.1021/acs.macromol.5b00857.

[ref33] Lu Y.; Wang Z.; An L.; Shi A.-C. Polymer Translocation Time. J. Phys. Chem. Lett. 2021, 12, 11534–11542. 10.1021/acs.jpclett.1c03202. 34806391

[ref34] Caraglio M.; Orlandini E.; Whittington S. Driven translocation of linked ring polymers through a pore. Macromolecules 2017, 50, 9437–9444. 10.1021/acs.macromol.7b02023.

[ref35] Chen Q.; Zhang L.; Ding M.; Duan X.; Huang Y.; Shi T. Effects of nanopore size on the flow-induced star polymer translocation. Eur. Phys. J. E 2016, 39, 1–6. 10.1140/epje/i2016-16109-3. 27853961

[ref36] Suma A.; Micheletti C. Pore translocation of knotted DNA rings. Proc. Natl. Acad. Sci. U. S. A. 2017, 114, E2991–E2997. 10.1073/pnas.1701321114. 28351979 PMC5393256

[ref37] Mair A.; Tung C.; Cacciuto A.; Coluzza I. Translocation of a globular polymer through a hairy pore. J. Mol. Liq. 2018, 265, 603–610. 10.1016/j.molliq.2018.06.009.

[ref38] Gao Q.-C.; Li Z.-Y.; Xu Y.-W.; Guo C.; Hou J.-X. Polymer translocation of linear polymer and ring polymer influenced by crowding. Mod. Phys. Lett. B 2019, 33, 1950318 10.1142/S0217984919503184.

[ref39] Weiss L. B.; Marenda M.; Micheletti C.; Likos C. N. Hydrodynamics and filtering of knotted ring polymers in nanochannels. Macromolecules 2019, 52, 4111–4119. 10.1021/acs.macromol.9b00516.

[ref40] Katkar H.; Muthukumar M. Single molecule electrophoresis of star polymers through nanopores: Simulations. J. Chem. Phys. 2018, 149, 163306 10.1063/1.5029980. 30384726 PMC6039299

[ref41] Nagarajan K.; Chen S. B. Flow-Induced Translocation of Star Polymers through a Nanopore. J. Phys. Chem. B 2019, 123, 7919–7925. 10.1021/acs.jpcb.9b07066. 31461281

[ref42] Dietrich-Buchecker C.; Sauvage J.; Kintzinger J. Une nouvelle famille de molecules: les metallo-catenanes. Tetrahedron Lett. 1983, 24, 5095–5098. 10.1016/S0040-4039(00)94050-4.

[ref43] Niu Z.; Gibson H. W. Polycatenanes. Chem. Rev. 2009, 109, 6024–6046. 10.1021/cr900002h. 19670889

[ref44] Rheaume S. N.; Klotz A. R. Nanopore translocation of topologically linked DNA catenanes. Phys. Rev. E 2023, 107, 024504 10.1103/PhysRevE.107.024504. 36932513

[ref45] Sean D.; Slater G. W. Highly driven polymer translocation from a cylindrical cavity with a finite length. J. Chem. Phys. 2017, 146, 054903 10.1063/1.4975091. 28178822

[ref46] Katkar H. H.; Muthukumar M. Single molecule electrophoresis of star polymers through nanopores: Simulations. J. Chem. Phys. 2018, 149, 163306 10.1063/1.5029980. 30384726 PMC6039299

[ref47] Guillouzic S.; Slater G. W. Polymer translocation in the presence of excluded volume and explicit hydrodynamic interactions. Phys. Lett. A 2006, 359, 261–264. 10.1016/j.physleta.2006.06.042.

[ref48] Izmitli A.; Schwartz D. C.; Graham M. D.; de Pablo J. J. The effect of hydrodynamic interactions on the dynamics of DNA translocation through pores. J. Chem. Phys. 2008, 128, 085102 10.1063/1.2831777. 18315085

[ref49] Thompson A. P.; Aktulga H. M.; Berger R.; Bolintineanu D. S.; Brown W. M.; Crozier P. S.; in’t Veld P. J.; Kohlmeyer A.; Moore S. G.; Nguyen T. D.; et al. LAMMPS-a flexible simulation tool for particle-based materials modeling at the atomic, meso, and continuum scales. Comput. Phys. Commun. 2022, 271, 108171 10.1016/j.cpc.2021.108171.

[ref50] Michaud-Agrawal N.; Denning E. J.; Woolf T. B.; Beckstein O. MDAnalysis: A Toolkit for the Analysis of Molecular Dynamics Simulations. J. Comput. Chem. 2011, 32, 2319–2327. 10.1002/jcc.21787. 21500218 PMC3144279

[ref51] McGibbon R. T.; Beauchamp K. A.; Harrigan M. P.; Klein C.; Swails J. M.; Hernández C. X.; Schwantes C. R.; Wang L.-P.; Lane T. J.; Pande V. S. MDTraj: A Modern Open Library for the Analysis of Molecular Dynamics Trajectories. Biophys. J. 2015, 109, 1528–1532. 10.1016/j.bpj.2015.08.015. 26488642 PMC4623899

[ref52] Humphrey W.; Dalke A.; Schulten K. VMD: Visual Molecular Dynamics. J. Mol. Graphics 1996, 14, 33–38. 10.1016/0263-7855(96)00018-5. 8744570

[ref53] Savitzky A.; Golay M. J. E. Smoothing and Differentiation of Data by Simplified Least Squares Procedures. Anal. Chem. 1964, 36, 1627–1639. 10.1021/ac60214a047.

[ref54] Edmonds C. M.; Hudiono Y. C.; Ahmadi A. G.; Hesketh P. J.; Nair S. Polymer translocation in solid-state nanopores: Dependence of scaling behavior on pore dimensions and applied voltage. J. Chem. Phys. 2012, 136, 065105 10.1063/1.3682777. 22360225

