# Supplementary material for: Constraints on Knot Insertion, Not Internal Jamming, Control Polycatenane Translocation Dynamics through Crystalline Pores
Source: Macromolecules. 2023 Apr 10;56(8):3238–45. doi: 10.1021/acs.macromol.2c02565 (PMC10141125; doi:10.1021/acs.macromol.2c02565)
Supplement: Supplementary file 1 — ma2c02565_si_001.pdf [file ma2c02565_si_001.pdf]

Supporting Information for "Constraints on Knot Insertion, Not Internal Jamming, Control Polycatenane Translocation Dynamics Through Crystalline Pores"

Zifeng Wang,[†,¶] Robert M. Ziolek,[*,‡,¶] and Mesfin Tsige[*,†]

†*School of Polymer Science and Polymer Engineering, The University of Akron, Akron, Ohio 44325-3909, United States*

‡*Department of Physics, King's College London, London, WC2R 2LS, United Kingdom*

¶*Z.W. and R.M.Z. contributed equally*

E-mail: robert.ziolek@kcl.ac.uk; mtsige@uakron.edu

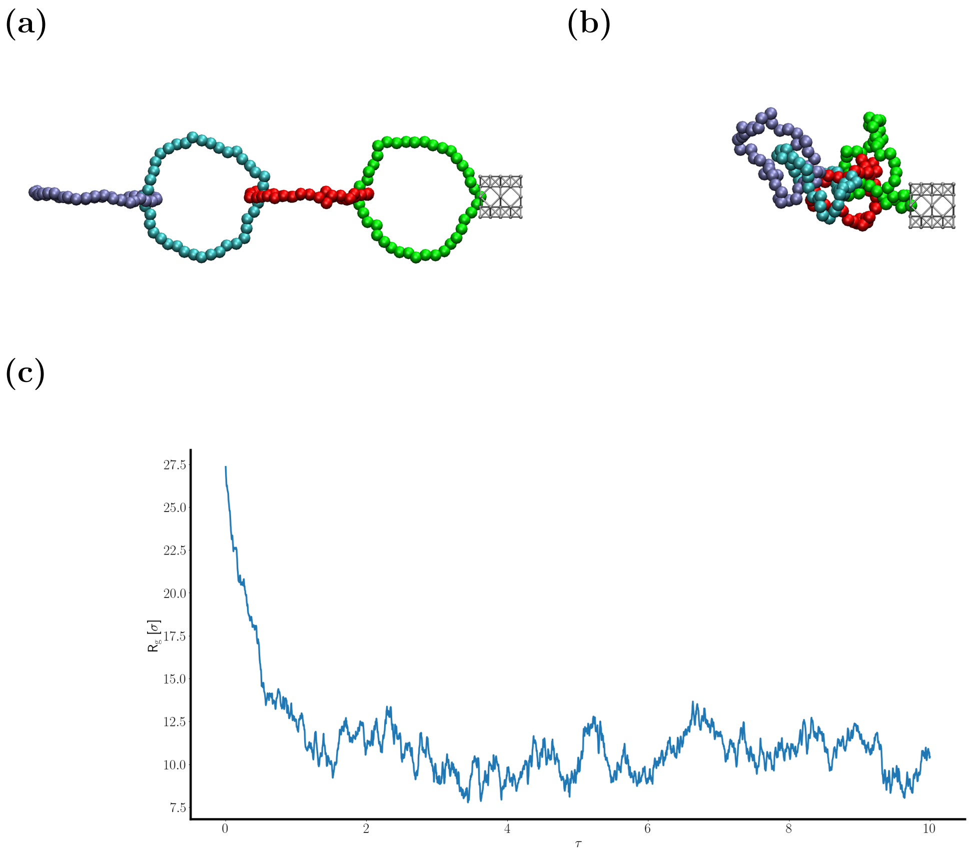

Figure S1: Initial polycatenane system conformation (a) before and (b) after initial structural equilibration procedure. (c) Indicative example of the radius of gyration of the polycatenane as a function of time during the equilibration process.

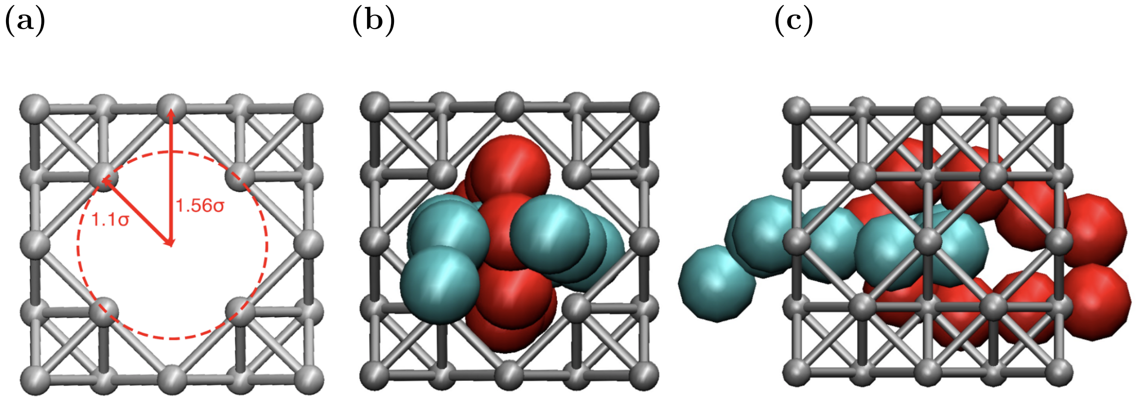

Figure S2: Snapshots showing the microstructure of the $r = 1.1$ pore interior. (a) Distances from the pore center to beads making up the pore interior. An example of a Hopf link passing through the pore (b) looking down the pore along the translocation axis and (c) observing the same snapshot perpendicular to the direction of translocation. Note that to improve visual clarity, the sizes of the pore beads and polycatenane beads are not proportional.

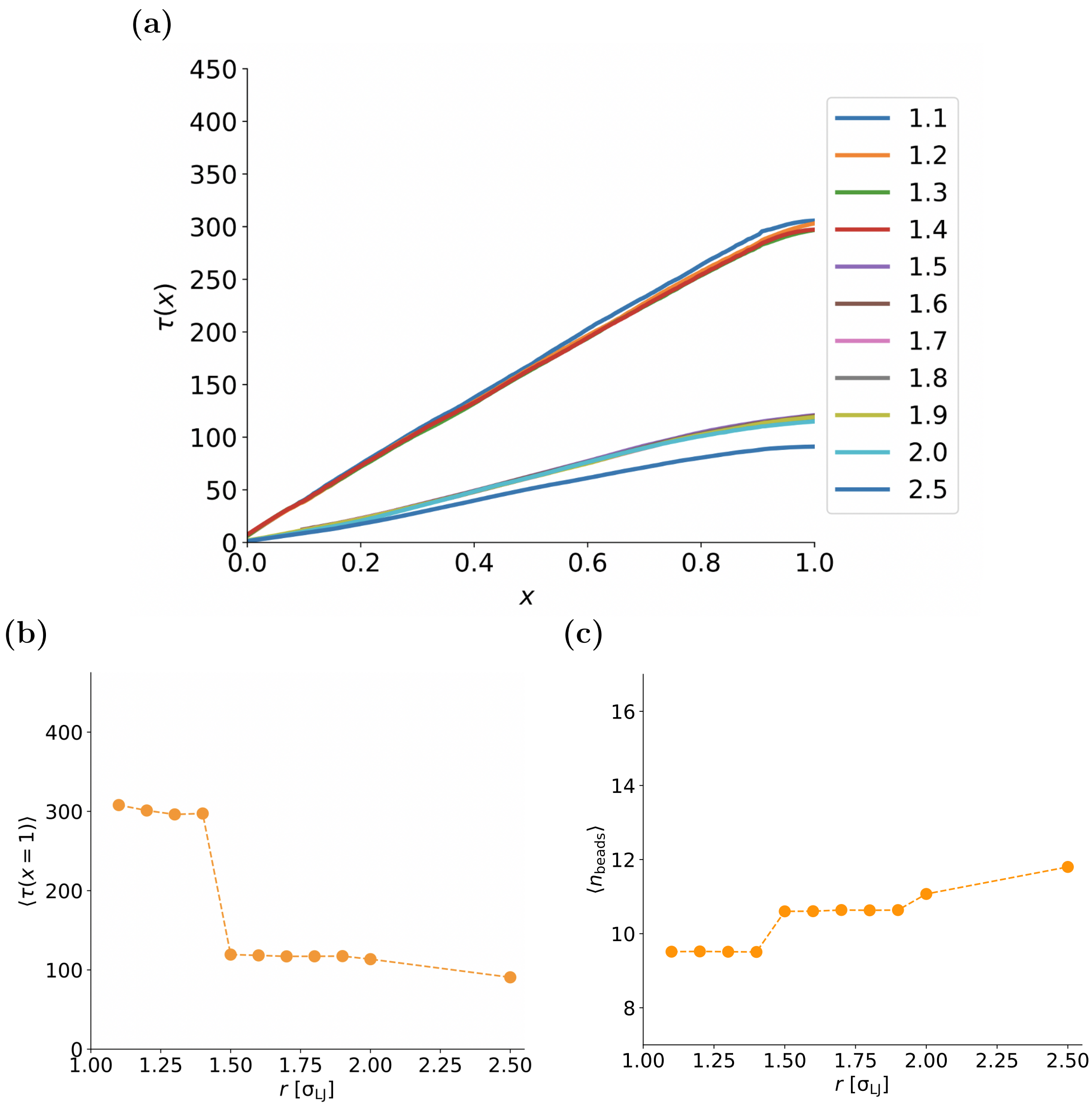

Figure S3: (a) Translocation profiles and (b) total translocation times of a ring polymer translocating through pores of different radii. (c) Number of beads within the pore as a function of the pore radius. N.B.: error bars representing the 95% confidence interval are smaller than the data point markers in all cases.

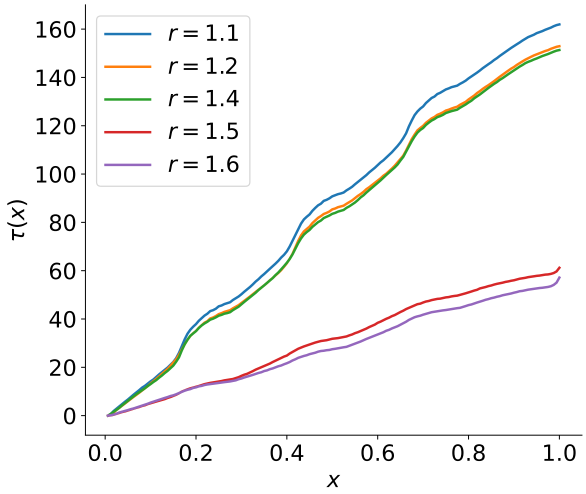

Figure S4: Translocation fraction (x) as a function of time (τ) for different pore sizes for $|\boldsymbol{f}^{\text{external}}| = 2\epsilon\sigma^{-1}$, i.e. an external driving force twice that used throughout the rest of this study. Note that the large drop off in translocation time still takes place between $r = 1.4$ and $r = 1.5$.

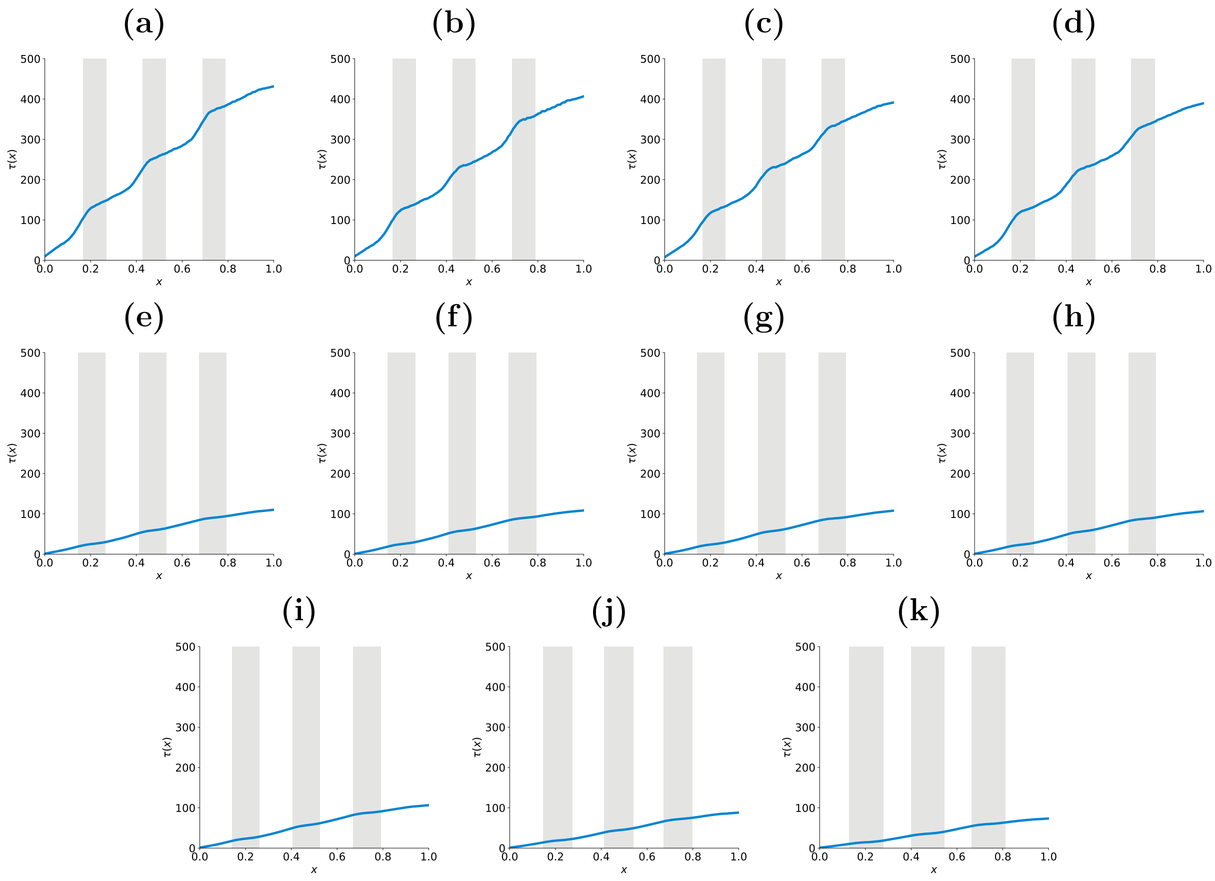

Figure S5: Polycatenane translocation profiles for the different pore radius sizes considered (these are the same as listed in Figure S1(a)), increasing in pore radius size from (a) to (k).

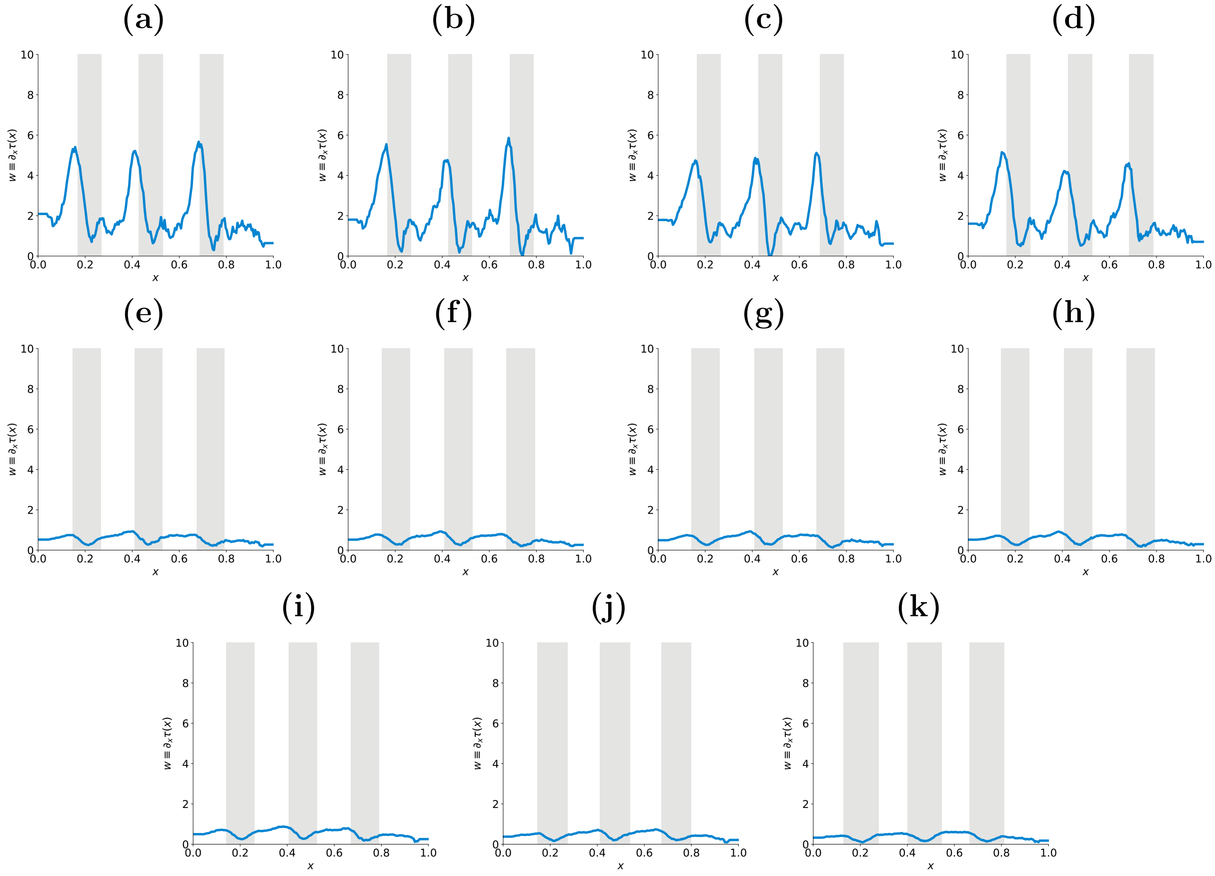

Figure S6: Polycatenane waiting time profiles for the different pore radius sizes considered (these are the same as listed in Figure S1(a)), increasing in pore radius size from (a) to (k).

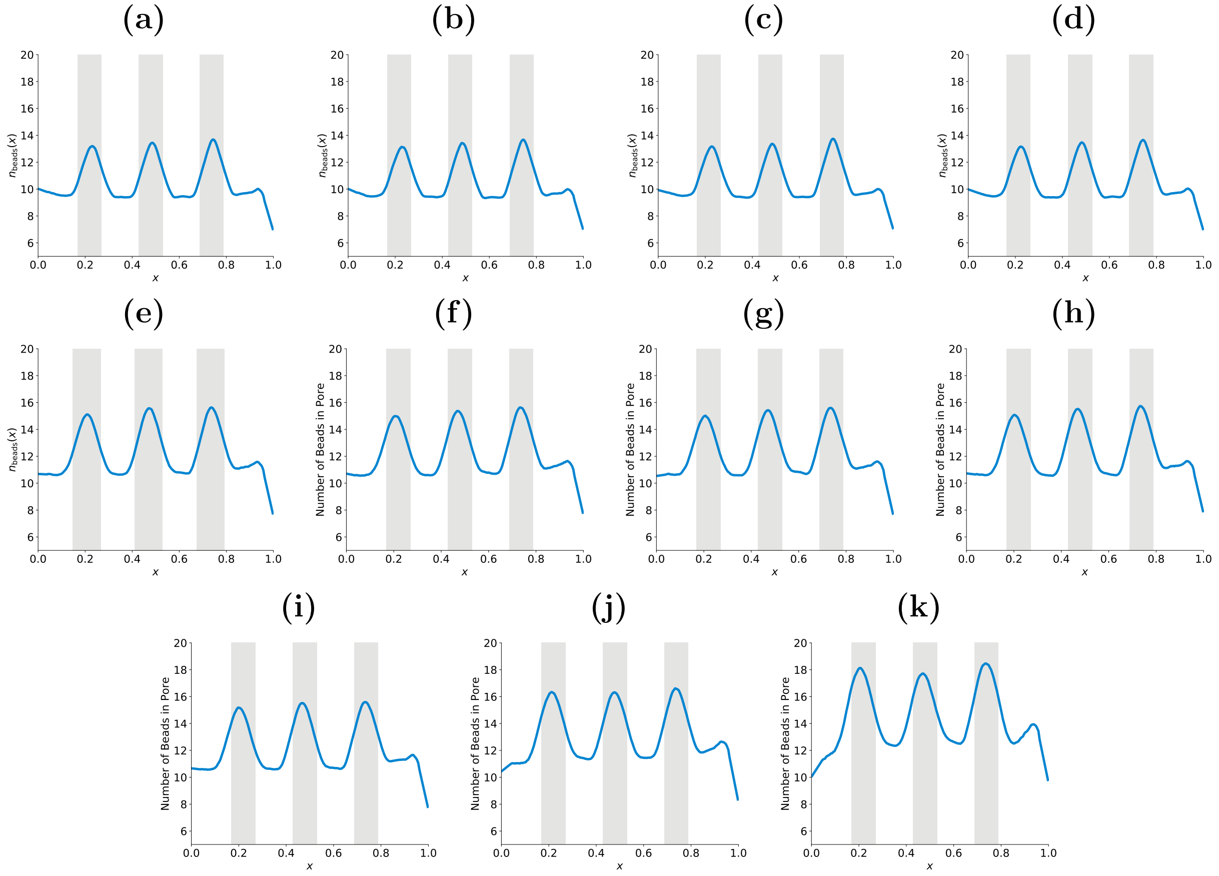

Figure S7: Average number of beads within the pore over the course of the polycatenane translocation process for the different pore radius sizes considered (these are the same as listed in Figure S1(a)), increasing in pore radius size from (a) to (k).

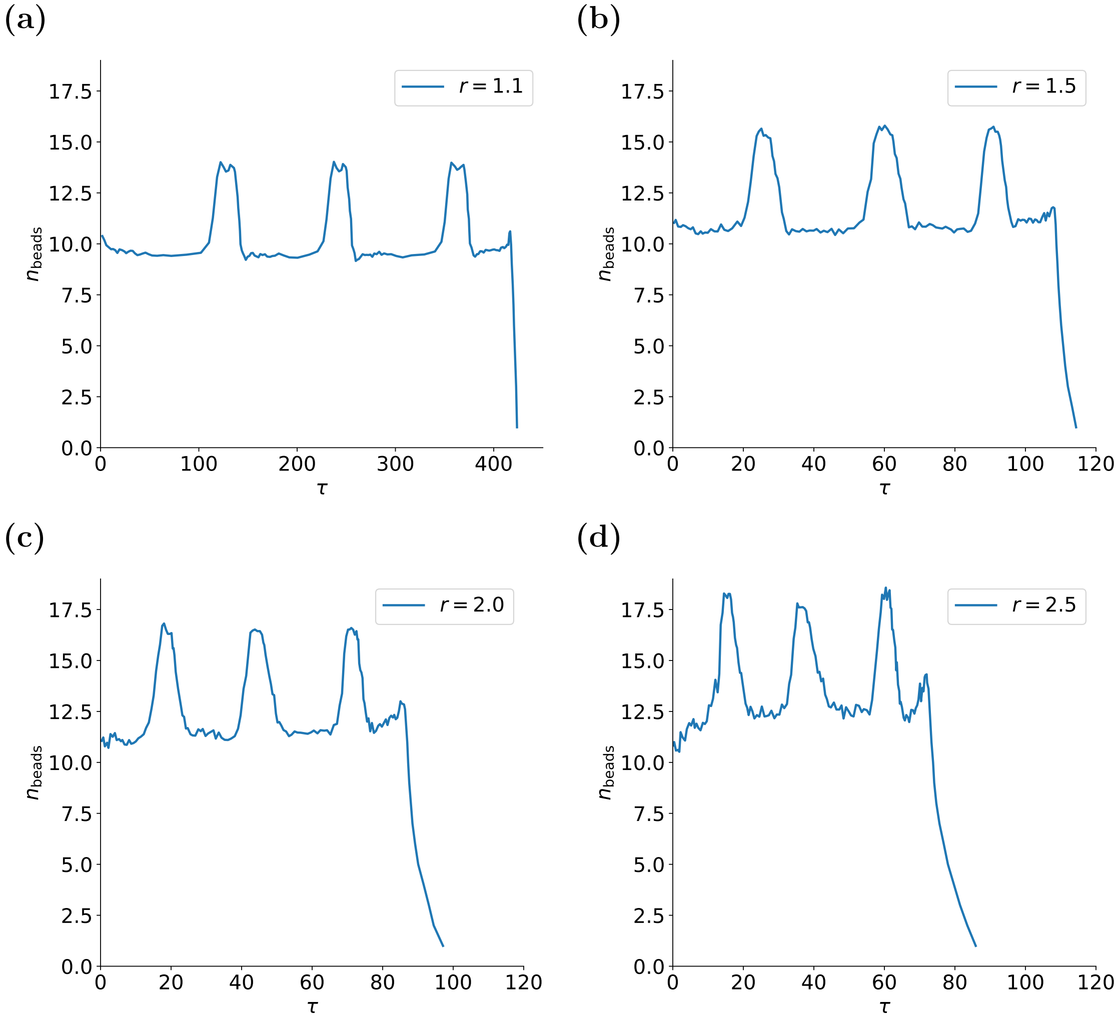

Figure S8: Number of beads within the pore as a function of time (τ) for different pore sizes (pore radius indicated by the legend in each plot). Note the difference in x-axis range between (a) and (b)-(d), which is enforced for clarity.

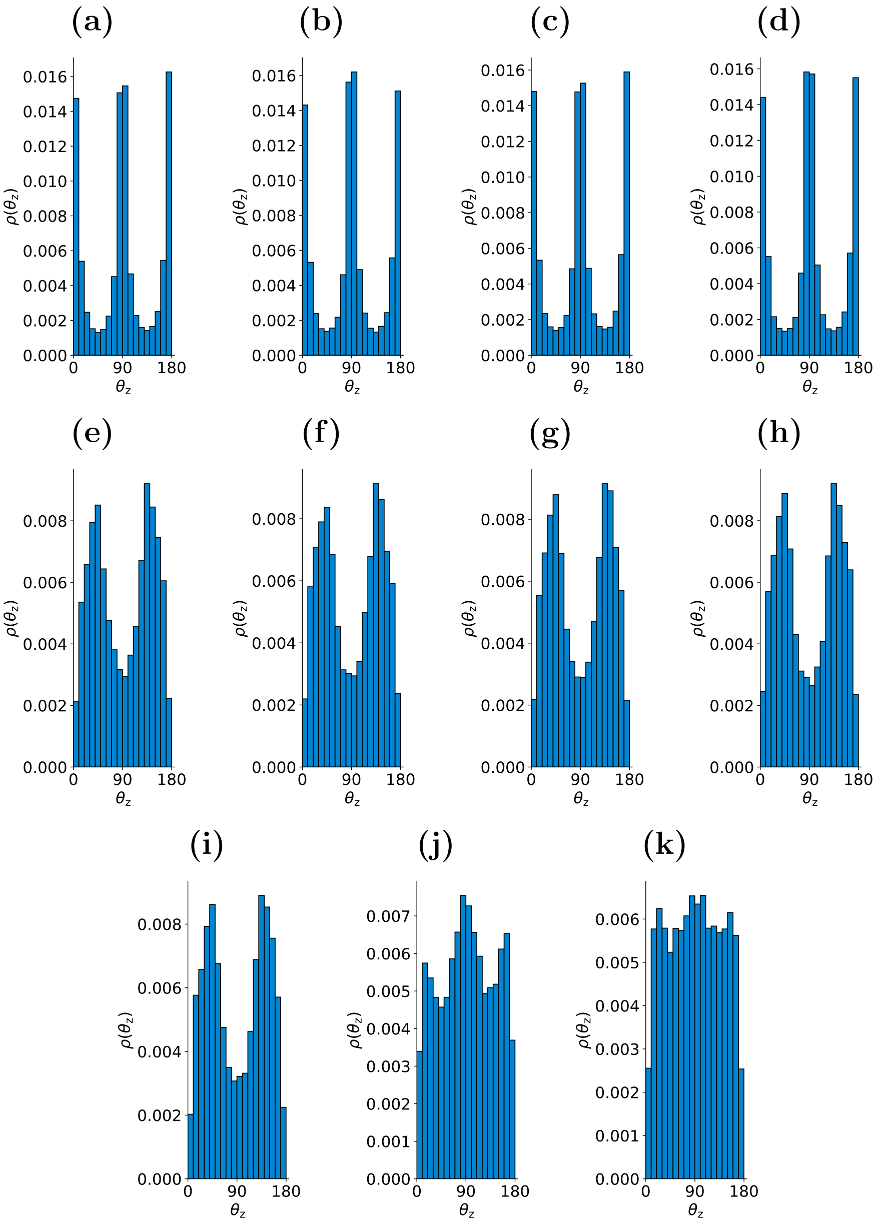

Figure S9: Polycatenane chain angle distributions within the nanopore. Note the symmetry in each distribution at $\theta_z = 90$, which reflects the internal symmetry of the pore and the SVD normal angle calculation.

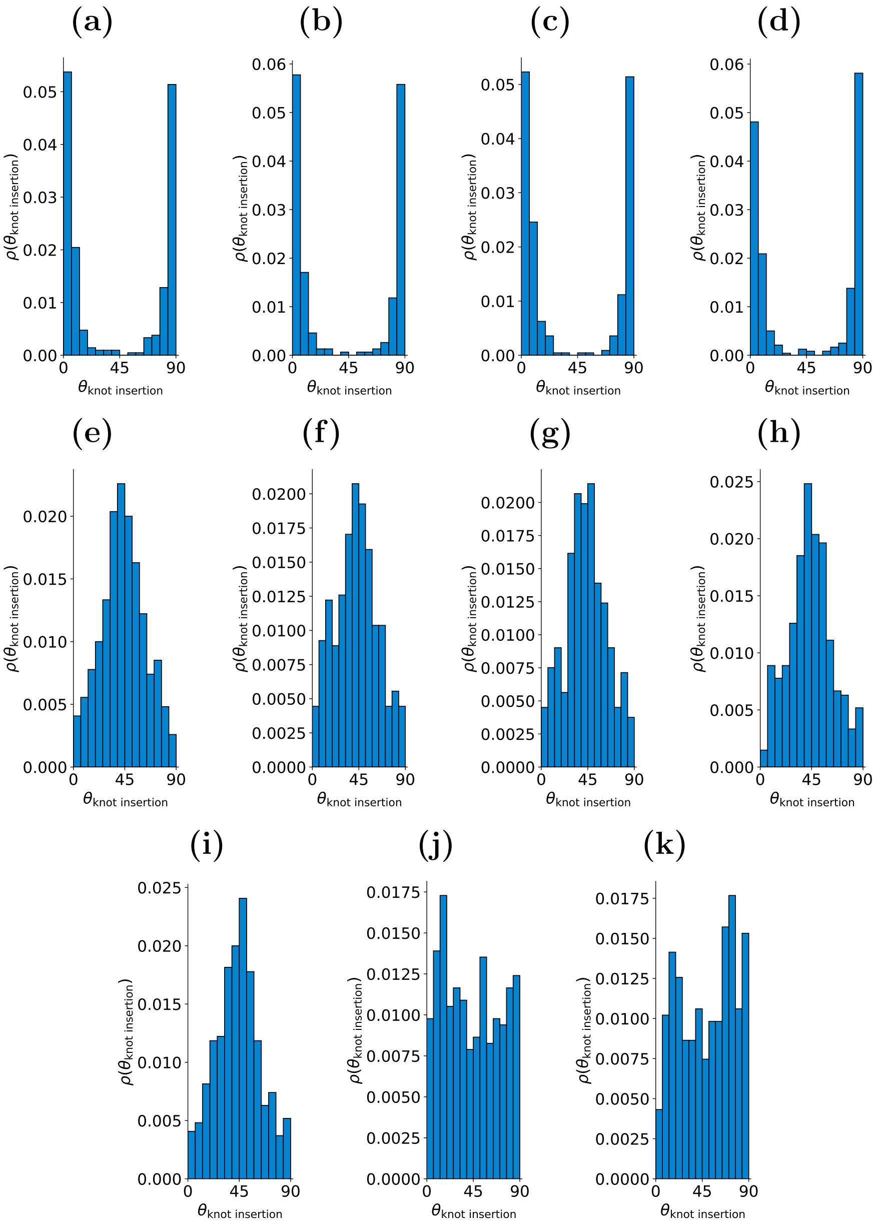

Figure S10: Polycatenane insertion angle distributions. These distributions are presented such that if $\theta > 90°$, then a conversion is applied ($\theta \rightarrow 90 - \theta$) to reflect this underlying symmetry and also to reduce noise in the statistics.
